# Each worm to his taste: some prefer to eat nettles – a giant gastric phytobezoar

**DOI:** 10.1002/ccr3.593

**Published:** 2016-06-03

**Authors:** Mahir Gachabayov, Abakar Abdullaev, Petr Mityushin, Timur Gilyazov

**Affiliations:** ^1^ Department of Abdominal SurgeryVladimir City Clinical Hospital of Emergency MedicineGorky Street, 6‐73600017VladimirRussia

**Keywords:** Gastric bezoar, gastrotomy, nettle, phytobezoar, *Urtica dioica*

## Abstract

Nettle consumption, as well as persimmon, orange, coconut etc. can lead to phytobezoar formation. Coke and cellulase‐resistant phytobezoars should be removed either endoscopically or surgically, depending on their dimensions. The treatment of choice for giant phytobezoars (more than 10 cm) is gastrotomy.

A 47‐year‐old male patient was admitted to the department of abdominal surgery with 6‐month history of epigastric pain and weight loss up to 15 kg. EGD revealed a giant gastric phytobezoar. Eating habits of the patient, coming from the south of Russia, included raw stinging nettle. Nonsurgical treatment including attempts of endoscopic mechanical fragmentation and diet coke ingestion appeared to be unsuccessful. The patient underwent laparotomy and gastrotomy with phytobezoar extraction as shown in Video S1. Postoperatively the patient recovered uneventfully.

Phytobezoars are formed of indigestible fibers of fruits and vegetables including tannin, lignin, and cellulose. Food types that lead to phytobezoar formation include persimmons, oranges, coconuts, apples, green beans, sauerkraut, figs, berries, Brussels sprouts, and potato peels 1, 2. The presented phytobezoar was formed of phloem fibers located in the stems of stinging nettle (*Urtica dioica*) which is eaten either raw or cooked in southern regions of Russia (Fig. 1). To the best of our knowledge, nettle bezoar has never been reported before. Predisposing factors of phytobezoar formation are patient‐related factors including gastric hypomotility and hypochlorhydria of any origin and edentulism 1 and food‐related factors such as high fiber diet 2. Clinical presentation and complications of bezoars correlate with their site, thus gastric bezoars lead to ulcer formation, gastrointestinal bleeding, and gastric outlet obstruction 3; while small bowel bezoars lead to intestinal obstruction 4. There are three treatment modalities for gastric phytobezoars: conservative, endoscopic, and surgical. Conservative methods include prokinetics, enzymes (cellulose, papain), diet coke, nasogastric coke lavage 3, 5. Endoscopic methods include endoscopic mechanical fragmentation 6, endoscopic suction removal with a large‐channel endoscope 7, endoscopic electrohydraulic lithotripsy 8, endoscopic laser lithotripsy 9, argon‐plasma coagulation, 6 and endoscopic injection of coke or enzymes 10. Giant phytobezoars (10 cm or more in diameter), as well as their surgical complications, such as perforation, obstruction, bleeding and spontaneous fistula formation, and unsuccessful non‐surgical treatment are the indications for surgery, which is commonly gastrotomy with bezoar removal via laparotomy or laparoscopic approach 11.

**Figure 1:**
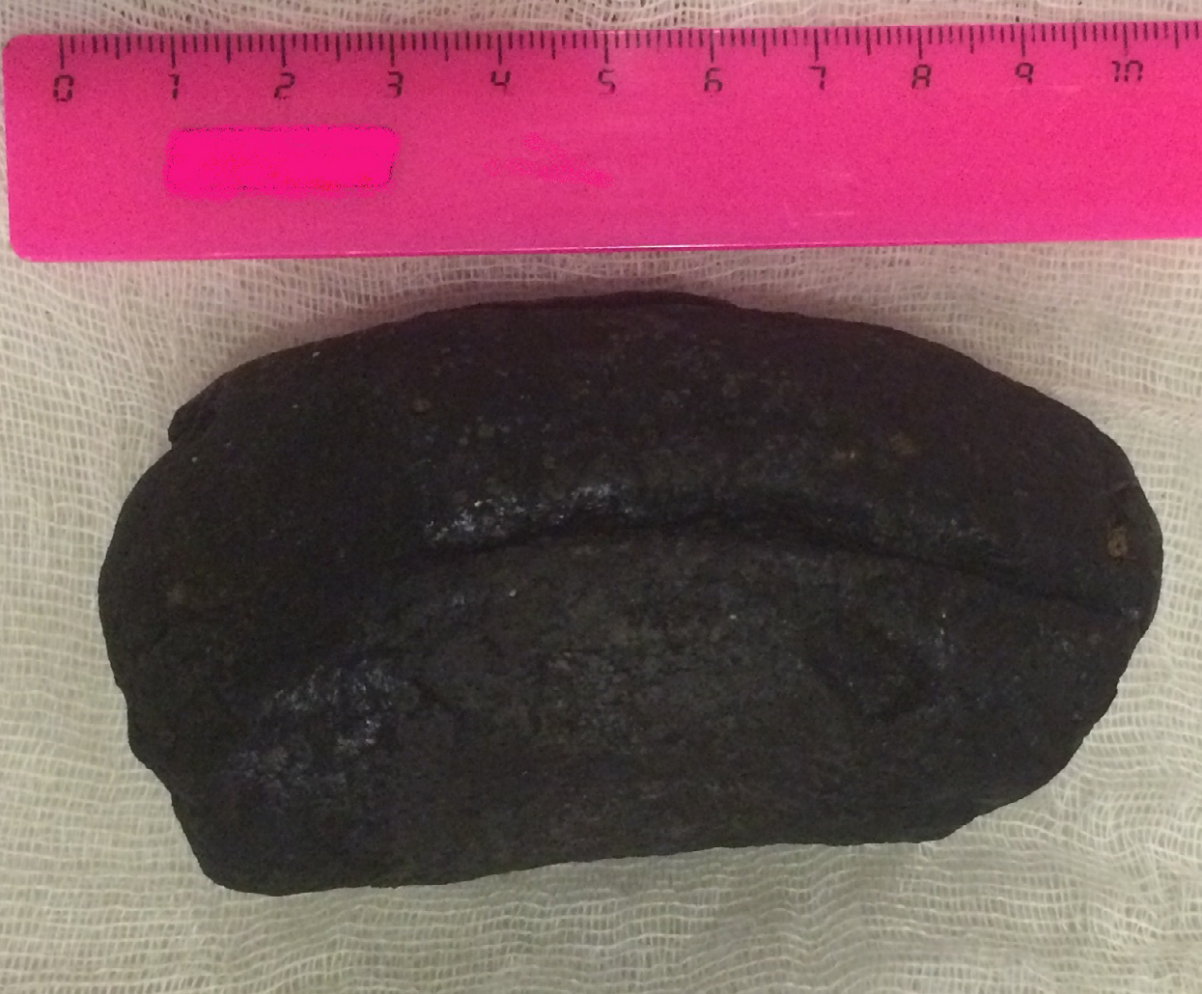
Surgically removed phytobezoar formed of phloem fibers of stinging nettle.

To conclude, since “each worm to his taste, some prefer to eat nettles” as expressed in Japanese proverb, healthcare professionals should be aware of phytobezoars and eating habits should be a part of history taking to identify high‐risk patients.

## Conflict of Interest

None declared.

## Supporting information


**Video S1.** Gastrotomy with phytobezoar removal.
